# Multiple Classifier System for Remote Sensing Image Classification: A Review

**DOI:** 10.3390/s120404764

**Published:** 2012-04-12

**Authors:** Peijun Du, Junshi Xia, Wei Zhang, Kun Tan, Yi Liu, Sicong Liu

**Affiliations:** 1 Department of Geographical Information Science, Nanjing University, Nanjing 210093, China; 2 Key Laboratory for Land Environment and Disaster Monitoring of State Bureau of Surveying and Mapping of China, China University of Mining and Technology, Xuzhou 221116, China; E-Mails: xiajunshi@126.com (J.X.); tankun@cumt.edu.cn (K.T.); rs_liuyi@163.com (Y.L.); allencong@163.com (S.L.); 3 Hebei Bureau of Surveying and Mapping, Shijiazhuang 050031, China; E-Mail: cumtwzh@163.com

**Keywords:** multiple classifier system, classifier ensemble, remote sensing, classification

## Abstract

Over the last two decades, multiple classifier system (MCS) or classifier ensemble has shown great potential to improve the accuracy and reliability of remote sensing image classification. Although there are lots of literatures covering the MCS approaches, there is a lack of a comprehensive literature review which presents an overall architecture of the basic principles and trends behind the design of remote sensing classifier ensemble. Therefore, in order to give a reference point for MCS approaches, this paper attempts to explicitly review the remote sensing implementations of MCS and proposes some modified approaches. The effectiveness of existing and improved algorithms are analyzed and evaluated by multi-source remotely sensed images, including high spatial resolution image (QuickBird), hyperspectral image (OMISII) and multi-spectral image (Landsat ETM+). Experimental results demonstrate that MCS can effectively improve the accuracy and stability of remote sensing image classification, and diversity measures play an active role for the combination of multiple classifiers. Furthermore, this survey provides a roadmap to guide future research, algorithm enhancement and facilitate knowledge accumulation of MCS in remote sensing community.

## 1. Introduction

Remote sensing images are widely used for land cover classification, target identification and thematic mapping from local to global scales owing to its technical advantages such as multi-resolution, wide coverage, repeatable observation and multi/hyperspectral-spectral records [1]. With the advancement of remote sensing data acquisition technology, remote sensing images can be acquired by various sensors, for example, hyperspectral imaging spectrometer, high resolution sensors, polarimetric synthetic aperture radar, *etc.* [2]. Under this situation, image classification techniques are exposed with new challenges to process the multi-source data and serve to multidisciplinary applications [2,3]. One effective solution is to generate a classifier ensemble by combining some individual classifiers, which is named as multiple classifier system (MCS) or classifier ensemble [4–10]. In the past twenty years, MCS has developed rapidly and been widely used in various fields such as pattern recognition, image processing and target identification. Besides, MCS has become a hot topic in the attractive series international workshops, mainly because of its capability to improve accuracy and efficiency [11–14].

MCS, which has been utilized by the remote sensing society recently, is viewed as one effective way to improve classification performance of remotely sensed imagery [3,4]. Many researchers investigated the scheme of combining several classifiers to generate a single result. Both theoretical and empirical studies indicate that a good MCS is one where individual classifiers are accurate and at the same time disagree on some different parts of the input space [15]. Two popular approaches for creating accurate ensemble are Bagging and Boosting [16,17]. Bagging uses bootstrap sampling to generate accurate ensemble [16]. Boosting is a general method of producing a very accurate prediction rule by combining rough and moderately inaccurate learner [17].

Some previous studies demonstrated that the simple majority vote of the classifier prediction is an effective strategy. Within this scheme, a pixel is classified as the class that was chosen by the majority of the individual classifiers. Foody et *al*. used simple majority vote rule to integrate multiple binary classifiers for the mapping of a specific class [7]. Benediktsson and Swain proposed a consensus theoretic classification approach and designed a hybrid consensus theoretic classification scheme [18,19]. The experimental results using hybrid consensus theoretic classification scheme outperform other statistical/neural network and consensus theoretic algorithms. Doan and Foody investigated the combination of soft classification methods for remote sensing image and found that classification combination could improve the accuracy [8]. Fauvel *et al* fused the classification results derived from a neural network and a fuzzy classifier using fuzzy sets theory, and the proposed method improved the classification accuracy compared to the separate use of different classifiers [20]. Smits focused on the family of MCSs based on dynamic classifier selection (DCS) and proposed a modification to dynamic classifier selection by local accuracy (DCS-LA) [9]. Experiments had shown that DCS-LA is a valid alternative method to majority vote.

In addition, MCS was also used for multi-source and hyperspectral remote sensing images. Briem *et al*. applied multiple classifiers (minimum Euclidean distance classifier, maximum likelihood classifier, conjugate-gradient back propagation, decision table, J48, 1R) to multisource remote sensing data (multispectral, synthetic aperture radar and topographical data sets) and demonstrated that Bagging, Boosting and consensus theory obtained higher overall user and producer accuracies than traditional classifiers [4]. Debeir *et al*. integrated spectral features, spatial and contextual information and experimented Bagging and subset selection algorithms with high resolution remotely sensed images, and the accuracy was higher than neural network and decision tree classifier [21]. Kumar *et al*. designed a hierarchical fusion strategy for classifying hyperspectral remote sensing image, and the classifier fusion method outperformed other traditional approaches, including maximum likelihood classifier (MLC) *etc.* [11]. Waske *et al*. fused Support Vector Machines (SVM) for classifying SAR and multispectral imagery from agricultural areas[22]. The presented approaches are superior to all other techniques, such as a standard SVM. Ceamanos *et al*. designed a classifier ensemble for classifying hyperspectral data [15]. Firstly, hyperspectral image is divided into several parts according to the similarity of spectral bands. Then, each part is used as the input of SVM classifier. Finally, all outputs are applied to be combined together by an additional SVM classifier. The results clearly demonstrated that the proposed SVM fusion scheme outperforms a standard SVM classifier. Owing to its technical superiorities, MCS is also applied to change detection from multi-temporal remote sensing images [23,24].

As mentioned above, the classification results to be combined should be diverse. If the classification results are identical or similar, it would not improve the accuracy by combining them. Therefore, diversity is a vital requirement for the success of ensemble, demonstrated by theoretical and empirical studies [25,26]. Kuncheva and Whitaker summarized the diversity measures in classifier ensembles [27]. A special issue called “Diversity Measure in Multiple Classifier System” published in Information Fusion journal indicates that diversity measure is an important research direction in MCS [28]. Petrakos *et al*. applied agreement measure in decision fusion level combination [29]. Foody compared the different classification results from three aspects (similarity, non-inferiority, difference) using hypothesis tests and confidence interval algorithms [30]. Brown *et al*. indicated that the diversity for classification tasks is still an ill-defined concept, and defining an appropriate diversity measures for MCS is still an open question [28]. So far, there are many existing diversity measures, but in the remote sensing literatures, they are rarely used and compared for remote sensing image classification by MCS. In conclusion, a successful MCS system depends to a large extent on the proper selection of diverse classifiers for incorporation [31]. Kuncheva claims that the choice of an appropriate ensemble strategy can improve the performance of MCS [32]. More details about diversity measures and classifier ensemble approaches can be seen in Section 2.

The objective of this paper is to give a review on the uses of MCS in remote sensing and the ideas of improving MCS approaches, including classifier ensemble approaches and diversity measures, and some modified algorithms. In order to demonstrate the effectiveness of MCS, the mentioned MCS approaches are experimented with three typical optical remotely sensed images: high spatial resolution image (QuickBird image), hyperspectral image (OMISII image) and multi-spectral image (Landsat ETM+ image).

The remainder of this paper is organized as follows. In Section 2, we introduce some common approaches of MCS, summarize the advances of MCS to remote sensing image classification, and propose some improved algorithms. Experimental results are presented in Section 3. Section 4 is the conclusion of this paper.

## 2. Multiple Classifier System for Remote Sensing Image Classification

### 2.1. MCS: A Primer

For remote sensing image classification, Giacinto *et al.* compared the performance of remote sensing classification approaches in different applications and found that no classifier could always perform well [33]. To alleviate this problem, MCS can provide the complementary information of the pattern classifiers and integrate the outputs of these pattern classifiers according to a certain combination approaches [5,34–36]. Therefore, it can outperform any individual classifier. In addition to MCS in the domain of classification, some concepts are similar to MCS, including multiple classifier combination [37,38], classifier ensemble [10,27,39], decision level combination [40,41], ensemble learning [42,43], *etc.*

We assume *M* classes exist on a remote sensing image, represented by *C*_1_ ∪ *C*_2_…*C*_i_ ∪ *C_M_, i* ∈ {1,2,…, *M*}. For hard classification, each pixel is allocated a specific class label *J, J* ∈ {1,2,…, *M*}. For soft classification, each pixel is depicted by a vector {*P*(1), *P*(2),…, *P(M)*}, where *P*(*i*) is the probability or membership of the pixel belonging to the *i^th^* class, or the abundance of the *i^th^* endmember within the pixel. When multiple classifiers are combined, both hard and soft classification results can be integrated.

**Figure 1.**
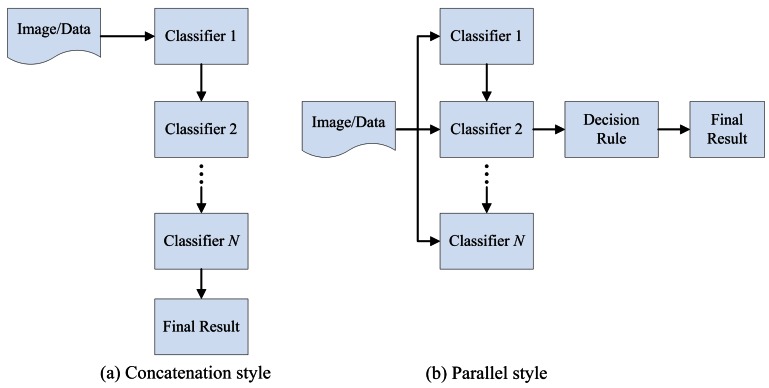
The combination style in MCS.

In order to introduce the MCS approaches clearly, three categories are adopted to summarize the classifier ensemble approaches:

1. Algorithms based on training sample manipulation. The most popular methods are Bagging and Boosting [16,17].
2. Concatenation combination. In a concatenation combination, the classification result generated by a classifier is used as the input into the next classifier. The results achieved through each classifier are similarly passed onto the next classifier until a result is obtained through the final classifier in the chain [31,44,45] (Figure 1(a)).
3. Parallel combination. In a parallel combination, multiple classifiers are designed independently without any mutual interaction and their outputs are combined according to certain strategies [44,45] (Figure 1(b)). The main design decision is the selection of a reprehensive ensemble approach. If the approach is well designed, the MCS can obtain the accurate result [31]. Some of the popular and successful ensemble approaches are majority voting, fuzzy integral, D-S evidence theory, *etc.*

Besides concatenation and parallel combination, hierarchical combination that combines both concatenation and parallel combination is also used [31]. But in this paper, we just focus on the concatenation and parallel combination.

As mentioned in Section 1, diversity of the classifier outputs is a vital requirement for the success of the ensemble. Diversity measures are often divided into parts: pairwise and non-pairwise [27]. Pairwise measures include kappa statistics, double fault, disagreement, *etc.*, and non-pairwise measures consist of entropy, weighted count of errors and correct results (WCEC), *etc.* [27,46]. When pairwise measures are used to calculate the diversity among more than two classification results, it adopts the average of the pairwise measures between each two classification results. The non-pairwise diversity measures consider all the classifiers together and calculate directly one diversity value for the ensemble [12]. In this paper, both pairwise and non-pairwise diversity measures, including kappa statistics, double fault, entropy, WCEC and a novel measure are reviewed.

The following sections describe the classifier ensemble approaches and diversity measures. Section 2.2 introduces Bagging and AdaBoost. Section 2.3 lists out a collection of parallel combination methods. Section 2.4 gives the basic idea of concatenation combination schemes. We proposed three modified concatenation combination approaches in Section 2.5. Section 2.6 presents five diversity measures, three of them are pairwise and the rest are non-pairwise.

### 2.2. Bagging and AdaBoost

Unlike statistical voting theory which is based on the assumption of independent data sources and uses all training samples only one time, Boosting and Bagging are exerted by manipulating training samples [16,17].

**Figure 2.**
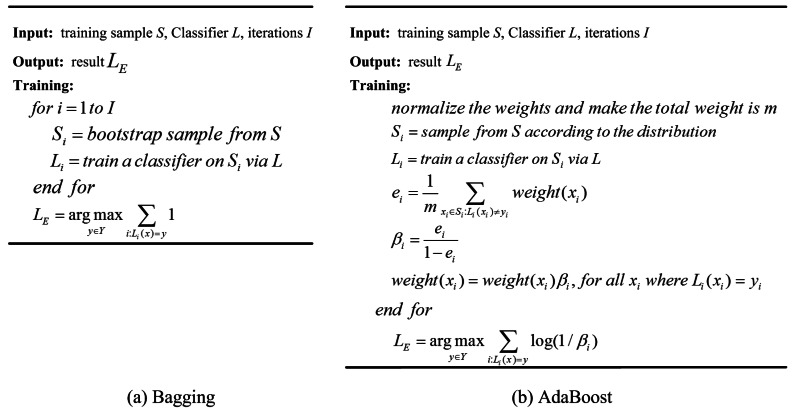
The pseudo-code of Bagging and AdaBoost [16,17,47].

Bagging is the abbreviation of bootstrap aggregating. In this algorithm, *n* samples are selected at random from a training set with *k* samples, and instructive iteration is exerted to create some different bags, and each bag is classified by vote to predict its class [16]. Boosting can process data with weights, and the weights of misclassified samples are increased to concentrate the learning algorithm on specific samples. Bagging has been shown to reduce the variance of the classification, while Boosting reduces both the variance and the bias of the classification. So in most cases, Boosting can produce more accurate classification results than Bagging. However, the computation time of Boosting is more than Bagging, and Boosting is sensitive to noise [47]. AdaBoost is the popular used approach of Boosting algorithms. The pseudo-code of Bagging and AdaBoost can be seen in Figure 2 [16,17,47]. In addition, there is a great variety of approaches drawn upon the basic idea of Bagging and Boosting. Detailed descriptions can be found from Wagging [48], Random Forest [49], Random Subspace [50], Logistic Boosting [51], MultiBoost [52], Rotation Forest [39], Rotboost [53], *etc.*

### 2.3. Parallel Combination

#### Majority or weighted vote

The most popular ensemble approach is the majority vote by which each individual classifier “votes” for the specific class, and the class that collects the majority votes is predicted by the ensemble [12]. By generalizing this approach, Xu *et al.* proposed a threshold plurality vote, which imposed a threshold on the number of votes to select the class [44]. The major problem of majority vote is that all the classifiers have the same “authority” regardless of their respective abilities to classify properly [54]. Therefore, the weighting methods are best suited for problems where the individual classifiers perform the same task. The simple one is that the decision of each classifier is weighted according to the estimated accuracy on the training set. Furthermore, Moreno-Seco *et al.* proposed three variant of weighted voting approaches: re-scaled weighted vote (RSMV), best-worst weighted vote (BWWV) and quadratic best-worst weighted vote (QBWWV) [54]. Among all voting algorithm tested in [54], each method has shown the different performance in different datasets. In this paper, we adopted the specific class accuracy generated by the individual classifier as the weights.

#### Bayesian average method

Bayesian combiners are used to linearly combine the probabilistic predictions of multiple classifiers by weighting their posterior probabilities [55]. It can be defined as follows:

(1) $$P(X\in C_{i}|X)=\frac{1}{k}\sum_{k=1}^{N}P_{k}(X\in C_{i}|X),i=1,2,\ldots ,M$$

Then, the class label is decided:

(2) $$L(X)=\arg\underset{i=1,\ldots ,M}{\max}(P(X\in C_{i}|X))$$

where *P_k_ (X* ε *C_i_*∣*X)* represents the probability of class *i* of pixel *X* in *k^th^* classifier, *M* is the total number of classes, *N* is the total number of classifiers. *L(X)* represents the class label of pixel *X*.

Other simple operators such as *Maximum, Minimum* and *Product* have been applied to combine the posterior probabilities [56]. On the basis of Bayesian framework, Kittler *et al.* provided a theoretical underpinning of many existing combination schemes based on the product and sum rule, showing that the sum rule is less sensitive to the errors of subsets of base classifiers [57].

#### Fuzzy Integral

Fuzzy integral is an effective information fusion algorithm based on the fuzzy measure [58,59]. Sugeno integral is a widely used fuzzy integral algorithm, in which fuzzy measure *g* is defined as a function in measurable space: *g*: Θ → [0,1], meeting the conditions as follows:

1. *g*(Φ) = 0, *g(S)* = 1,
2. If *A, B* ∈ Θ, and A ⊆ B ⇒ *g* (*A)* ≤ *g*(*B*),
3. If ${\left\{A_{i}\right\}}_{i}^{\infty }$ is a incremental measurable set, then *g* (lim*_i_*_→∞_) = lim*_i_*_→∞_ *g*(*A_i_*).

Fuzzy measure *g*λ was introduced in Sugeno integral.

For any *A, B* ∈ Θ, *g(A* ∪ *B)* = *g(A)* + *g(B)* + λ*g(A)g(B)*, λ > −1.

Suppose that (*S, R, g*) is the fuzzy measurement space, and f : *S* → [0,1] is a measurable function on *R*. When *S* = *s*_1_, *s*_2_, …, *s_N_* is a finite set and meets the condition of 1 ≥ *f*(*s*_1_) *≥ f*(*s*_2_) … ≥ *f*(*s_n_*) ≥ 0, the fuzzy measurement can be calculated as the follows:

(3) $$g(A_{1})=g(\{s_{1}\})$$

(4) $$\begin{array}{l} g(A_{i})=g(\{s_{1}\})+g(A_{i-1})+\lambda g(\{s_{1}\})g(A_{i-1}),1<i\leq N \end{array}$$

where *A_i_* = {*s*_1_, *s*_2_, …, *s_i_*} is the classifier ensemble, and *N* is the amounts of classifiers.

Sugeno integral is calculated in the following:

(5) $$E_{j}=\int_{A}f_{j}(s_{i})g(A_{i})=\underset{i\in [1,m]}{\sup}\text{min}\{f_{j}(s_{i}),g(A_{i})\}$$

When the member classifiers are more than two, the values of λ corresponding to each class is calculated as the following equation:

(6) $$\lambda +1\;=\prod_{i=1}^{N}\left(1+g(\{s_{i}\})\right)$$

In multiple classifier combination, *f_j_*(*s_i_*) is defined as the probability of the *i^th^* classifier to the *j^th^* class in soft classification, or the accuracy of the *i^th^* classifier to the *j^th^* class in hard classification. We utilized the latter one, hard classification in this paper.

The final classification result is the class label corresponding to the maximum fuzzy integral value:

(7) $$L=\arg\underset{j=1,\ldots ,M}{\max}(E_{j})$$

Moreover, Ben Adballah *et al*. proposed a local fuzzy integral approach, called context extraction for local fusion with fuzzy integral (CELF-FI) for fusing different classifiers outputs [60]. This method based on a novel objective function that combines context identification and multi-algorithm fusion criteria into a joint objective function. This objective function consists of two terms: the first is designed to produce compact clusters, called contexts, and the second to produce Sugeno measures for fuzzy integral fusion for each context [60].

#### Consensus Theory

Consensus theory is suitable for the combination of classifiers with probability to specific classes as their outputs [18,19]. Usually logarithmic and linear consensus models are used in the following two ways:

(8) $$T_{j}(X)=\prod_{i=1}^{N}{\left(p_{i}(C_{j}|X)\right)}^{\lambda ij}$$

(9) $$T_{j}(X)=\sum_{i=1}^{N}(p_{i}(C_{j}|X))\lambda_{\text{ij}}$$

Each classifier is viewed as an expert with the membership or probability of the pixel belonging to every class as its output. *T_j_(X)* is the probability the pixel *X* being assigned to the *j^th^* class, and p*_i_ (C_j_*∣*X)* is the probability of pixel *X* being assigned to the *j^th^* class by the *i^th^* classifier, and λ*_ij_* is the weight. The simplest approach of the weighting scheme consists in giving all the individual outputs equal weights. Furthermore, the weights can be defined to not only weight the individual sources but also the individual classifiers [5]. Thus, we select the specific class accuracy as the weights. It means that λ*_ij_* represents classification accuracy of the *i^th^* classifier to the *j^th^* class in this paper.

The final classification result is the class label corresponding to the maximum value of *T_j_(X), j* = 1,…, *M*:

(10) $$L(X)=\arg\underset{j=1,\ldots ,M}{\max}(T_{j}(X))$$

#### D-S Evidence Theory and its improvement

D-S evidence theory, which assigns probability to the sets, is able to handle the uncertainty caused by unknown factors [12]. It uses discrimination framework, confidence function, likelihood function and probability allocation function to represent and process knowledge. Suppose that Θ = *C*_1_, *C_2_*,…, *C_i_*… *C_M_* is the discrimination framework and *M* is the number of classes, therefore basic probability allocation function *m* is a function from 2^Θ^ to [0,1] and it meets the requirement of:

(11) $$\{\begin{array}{r} m(\phi )=0\\ \sum_{A\subseteq \Theta }m(A)=1 \end{array}$$

If there are two or more different evidences, orthogonal sum can be used to combine those evidences. Assume that *Z*_1_, *Z*_2_,…, *Z_n_* are the probability allocation functions corresponding to evidence *F*_1_, *F_2_*,…, *F_n_*, and their orthogonal sum *Z* = *Z*_1_ ⊕ *Z_2_* … ⊕ *Z_n_* is:

(12) Z(ϕ)=0

(13) $$Z(A)=\frac{1}{K}\sum_{\cap A_{i}}\prod_{1\leq i\leq n}Z_{i}(A_{i})$$

(14) $$K=\sum_{\cap A_{i}\neq \Phi }\prod_{1\leq i\leq n}Z_{i}(A_{i})$$

When various evidences are inconsistent or contradictory from each other, the result may be unreasonable. In order to alleviate this problem, an improved evidence theory algorithm was proposed and experimented in [61], and it proved that the modified method was superior to traditional method while the evidences with high contradiction and inconsistency. For remote sensing image, different classifiers may generate different classified labels, resulting in the generation of evidence with high contradiction, so the improved evidence theory ensemble approach may improve the performance. The detailed equations are detailed as follows [61]:

(15) $$Z(A)\sum_{A_{i}\in F_{i}}Z_{i}(A_{i})Z_{2}(A_{2})\ldots Z_{n}(A_{n})+K\times \in \times \frac{1}{n}\sum_{i=1}^{n}Z_{i}(A)$$

where:

(16) $$\tilde{k}=\frac{2}{n(n-1)}\sum_{i<j}\sum_{A_{i}\cap A_{j}=\Phi ,A_{i}\in F_{i},A_{j}\in F_{j}}Z_{i}(A_{i})Z_{j}(A_{j})$$

(17) $$\in =\exp^{-\tilde{k}}$$

where *ε* is the confidence of evidence, *k̃* is the average of contradiction level between two evidences, and *K* is the total contradiction level of all evidences. This evidence combination method can reduce the limitations caused by evidence inconsistency. For multiple classifier combination of remote sensing, the result of each classifier can be viewed as a piece of evidence. Probability allocation function can be represented by the classification accuracy of specific class. For example, if a pixel belongs to the *i^th^* class in a classifier, the basic probability is *m*(C*_i_*) = *P_i_, m*(Θ) = 1 − *P_i_*, where *P_i_* is the accuracy of the *i^th^* class by the specific classifier. After the completion of evidence combination, the class with maximum evidence is selected as the final result.

#### Dynamic Classifier Selection [9,62]

Smits applied dynamic classifier selection and improved models to multiple classifier combination for remote sensing images [9]. In this method, MCS are addressed that use estimates of each individual classifier's local accuracy in small regions of feature space surrounding an unknown test sample [9]. The local regions are defined by the *k*—nearest neighbors (*KNN*). The drawback of this approach is the section of *k*, which is rather heuristic and subjective an exercise, and the classification result is sensitive to the choice of *k* [9,63,64].

The concept of local accuracy assumes that classes maintain a certain continuity in the feature space and neighboring pixels are expected to maintain a stronger relationship than pixels that are further away [9]. Dudani suggested that using distance-weighted *k*—NN can improve performance, especially with small training sample [65]. Suppose that *d_k_, d_t_, d_m_* are the distances of the *k^th^, t^th^* and *m^th^* samples to the pixel in the decreasing sort, the weight of the *t^th^* sample is:

(18) $$\omega_{t}=\{\begin{array}{ll} \frac{d_{k}-d_{t}}{d_{k}-d_{m}} & d_{k}\neq d_{m}\\ 1 & \text{otherwise} \end{array}$$

The local classification accuracy of the *j*^th^ classifier is:

(19) $$\begin{array}{ll} L^{j}=\begin{array}{l} \frac{1}{k}\cdot \sum_{\theta (X_{t})C_{j}(X_{t})}\omega_{t}, \end{array} & 1\leq t\leq k \end{array}$$

where *θ(X_t_)* is the class label of the *t^th^* neighboring sample, *C_j_(X_t_)* is the assigned class label of the *j^th^* classifier to the *t^th^* sample. Finally, the label of the classifier with the biggest local accuracy is selected as the final classification result.

#### Differences between these methods

In addition, according to the individual output, classifier ensemble approach in a parallel combination can be divided into three levels: abstract level, rank level and measurement level [66]. The abstract level combination methods are applied when each classifiers outputs a unique label [66]. Rank level makes use of a ranked list of classes where ranking is based on decreasing likelihood. In the measurement level, probability values of the classes provided by the each classifier are used in the combination. Therefore, majority/weighted vote, fuzzy integral, evidence theory and dynamic classifier selection belong to the abstract level combination methods, while others are measurement level methods. Table 1 summarizes the classifier ensemble approaches in parallel combination. Weighted vote, fuzzy integral, Dempster–Shafer evidence theory and consensus theory require validation set to calculate the weights while other methods do not. Dynamic classifier selection calculates the distance between the samples so it requires the original image. And the computation time of dynamic classifier selection is more expensive than other approaches.

### 2.4. Concatenation Combination

In the original concatenation combination approach, the classification result generated by a classifier is used as an input into the next classifier [31]. The results obtained through each classifier are similarly passed onto the next classifier until a result is obtained through the final classifier in the chain, which can be clearly shown in Figure 1(a). In this structure, decisions could be first made by a subset of the committee classifiers and their conclusions are then passed to another subset of classifiers. The construction of this algorithm is usually required to improve the accuracy of the entire system and to incorporate error checks [31]. Two potential ways to improve concatenation combination are enhancing the input diversity of the final combiner, and selecting an advanced combination algorithm.

### 2.5. Modified Concatenation Combination Approaches

**Figure 3.**
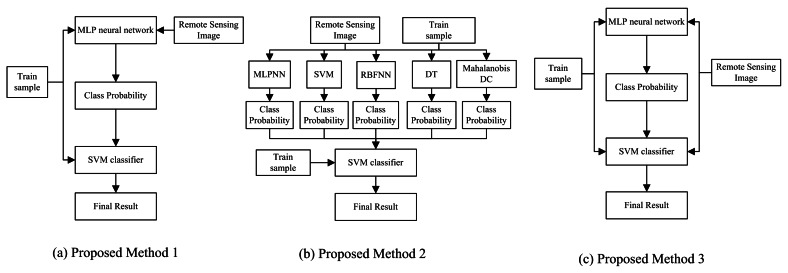
The structure of concatenation combination.

On the basis of the previous improvement ideas, we propose three modified concatenation combination approaches of MCS in this section. The overall architecture is given in Figure 3. All the proposed schemes contain two training stages. Among the three methods, SVM classifier is selected as the second classifier because of its satisfactory capability to deal with the classification problem [67,68].

Proposed method (1) is the original concatenation combination approach. Multi-layer perceptron neural network (MLPNN) is assigned as the first level classifier. The class probabilities generated by MLPNN are defined as the input of the second level. In order to enhance the diversity in concatenation combination approach, the class probabilities generated by the several classifiers are combined as the input in method (2). The class probabilities generated by MLPNN, Radial Basis Function neural network (RBFNN), C4.5 decision tree (DT), Minimum Mahalanobis distance classifier (MMDC) and SVM are treated as the input to train the new SVM classifier. Similar to method (2), method (3) adopts the input derived from the different source to enhance the diversity. Original remote sensing image and the class probabilities produced by MLPNN are defined as the input of the second level in method (3).

### 2.6. Diversity Measures

Kappa statistics, double fault, entropy and WCEC are used in this paper, and a novel measure is proposed for remote sensing based on the improvements in general MCS.

#### Kappa statistic

This measure is calculated by the following equation [29]:

(20) $$k=\frac{2(N^{11}N^{00}-N^{10}N^{01})}{p_{1}q_{2}+p_{2}q_{1}}$$

where *N*^11^ represents the samples correctly classified by both classifiers, *N*^10^ is the samples correctly classified by the first classifier and incorrectly by the second classifier, *N*^01^ represents the samples incorrectly classified by the first classifier and correctly by the second classifier, *N*^00^ is the samples incorrectly classified by both classifier. *p*_1_ represents the samples correctly classified by the first classifier (it equals *N*^11^ plus *N*^10^), *p*_2_ represents the samples correctly classified by the second classifier (it equals *N*^11^ plus *N*^01^), *q*_1_ represents the samples incorrectly classified by the first classifier (it equals *N*^01^ plus *N^00^), q_2_* represents the samples incorrectly classified by the second classifier (it equals *N*^10^ plus *N*^00^).

It not only gives a measure of the degree of agreement but also has a test associated with itself to check if the apparent agreement can be attributed to chance only In addition, we also use a new kappa statistic *k*′ and the final value is the average of the class kappa statistic.

#### Double fault

Gacinto and Roli proposed the double-fault measure to select classifiers that are least related from a pool of classifiers. This diversity decreases when the value of the double-fault measure increases [55]. This index is calculated by:

(21) $$DF=\frac{N^{00}}{N}$$

where *N* represents the number of sample.

#### Entropy

Entropy is calculated in the following [27]:

(22) $$E=\frac{1}{N}\sum_{m=1}^{N}\frac{1}{L-\left\lceil L/2\right\rceil }\text{min}\left\{l({\text{z}}_{m}),L-l({\text{z}}_{m})\right\}$$

where *L* is the number of classifiers, *N* is the number of sample, ⌈⌉ rounds a number to the nearest integer. *l*(***z****_m_*) is the number of samples correctly classified by the classifiers **z**.

This measure is based on the assumption: if the results of ⌈*L/2*⌉ is the same, the diversity has the biggest value. The range of this measure is 0 to 1, in which 1 represents the biggest diversity value and 0 represents the lowest diversity value.

#### Weighted count of errors and correct (WCEC)

*WCEC* is also proposed by [46]. Compared to kappa statistics, WCEC not only gives a measure of the correctness of classifiers but also consider the diversity between two classifiers.

(23) $$\text{WCEC}=\frac{N^{11}+\frac{1}{2}\left(N^{01}+N^{10}\right)-N_{\text{diff}}^{00}-5N_{\text{same}}^{00}}{N}$$

where ${\text{N}}_{\text{diff}}^{00}$ represents the different errors occurring in both classifiers, ${\text{N}}_{\text{same}}^{00}$ represents the same errors occurring in both classifiers.

#### Disagreement-accuracy measure

Combining similar classifiers does not make too much sense as the results will not be improved [31]. If the sample is classified correctly or incorrectly in the classifiers, the final result is not improved. In order to consider both the classification accuracy and disagreement among the classification results comprehensively, we propose a new diversity measure, namely, disagreement and accuracy measure (*DA*). Firstly, we have made the classification results in descending order according to the overall accuracy. Then *DA* was calculated by the contribution of the classifiers to each sample. It can be defined as the following:

(24) $$DA=\frac{1}{N}\sum_{i=1}^{N}\sum_{j=1}^{L}\frac{P_{i}(z_{j})}{2^{j-1}}$$

where *N* is total number of samples, *L* is the number of classifiers, *P_i_*(**z***_j_*) is the specific class accuracy of correctly classified sample *i* by the classifier **z***_j_*. This measure highlighted the contribution of classifier with high overall accuracy to the sample. That means if a sample is classified correctly by the classifier which has the highest overall accuracy, the contribution of the subsequent classifier to this sample should be decreased.

## 3. Experiments and Discussions

In order to evaluate the effectiveness and performance of MCS to remote sensing image classification, the aforementioned existing and modified methods are experimented in the three types of remote sensing images: high spatial resolution QuickBird image, airborne hyperspectral image, and medium-resolution multispectral Landsat ETM+ image.

The classifiers used in an ensemble should generally be accurate but different. Based on the survey to existing pixel-based classification algorithms by [3] and many suggestions on the selection of classifiers by [7], we have chosen nine classifiers to be used, including Maximum Likelihood Classifier (MLC), Minimum Euclidean Distance Classifier (MEDC), Minimum Mahalanobis distance classifier (MMDC), support vector machine (SVM), MLP neural network (MLPNN), RBF neural network (RBFNN), spectral angle mapper (SAM), J48(C4.5) decision tree classifier (DTC) and simplified fuzzy H-ARTMAP (SFH-ARTMAP). The descriptions of classifiers can be detailed in [68–75].

MLC, MDC, SAM, SVM in ENVI 4.5 and Bagging, AdaBoost, J48DT, MLPNN, RBFNN in Weka 3.6.3 software are used, while SFH-AREMAP is implemented in MATLAB 6.5 software [72]. The other classifier ensemble approaches and diversity measures are all implemented in ENVI+IDL software.

In this paper, we mainly focus on the final results of MCS, so we adopt the default parameters in the above classifiers. The iterations of AdaBoost and Bagging are 10. The different classifier ensemble algorithms are experimented in the three typical images, but the diversity measures are only experimented on QuickBird image.

### 3.1. Experiment 1: High Spatial Resolution Image Classification

**Figure 4.**
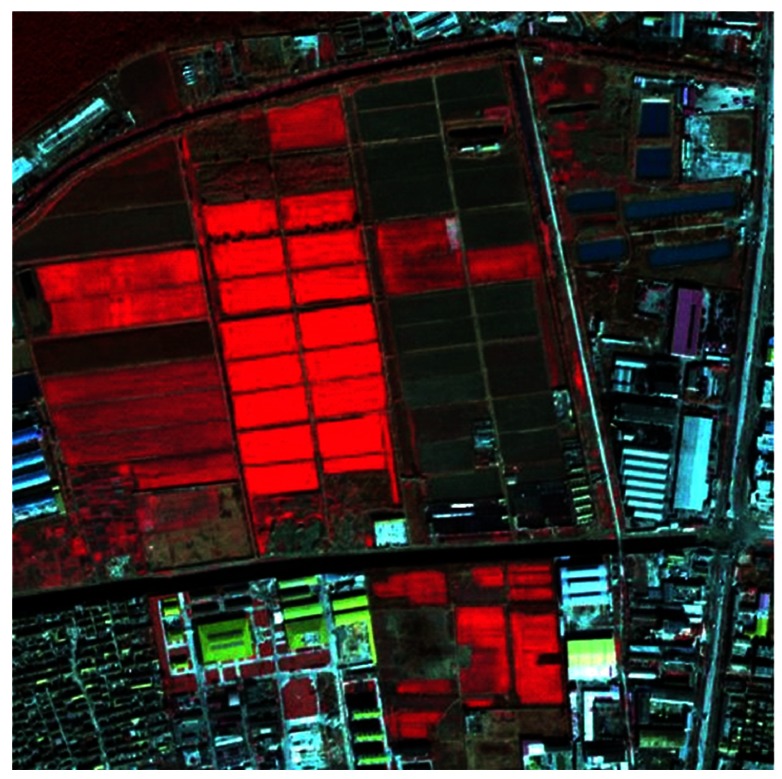
False color composite of QuickBird remote sensing image.

QuickBird multispectral image (four bands of multi-spectral, spatial resolution: 2.44 m) is used as the example of high spatial resolution remote sensing image. The image size is 500 × 500. The study area is located in the northern suburb of Xuzhou city along the urban and rural connecting areas. Through the image analysis and field works, the image is classified into five land cover types: water, built-up area, green areas, vegetation and barren soil. Figure 4 is the false color composite image (R: Band 4, G: Band 3, B: Band 2) of the study area.

**Figure 5.**
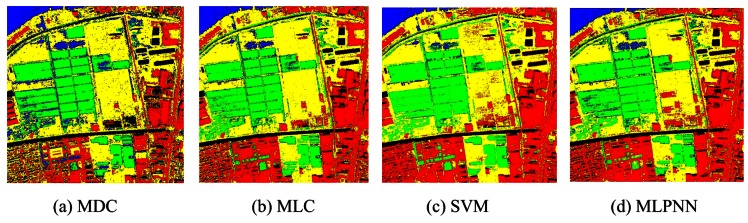

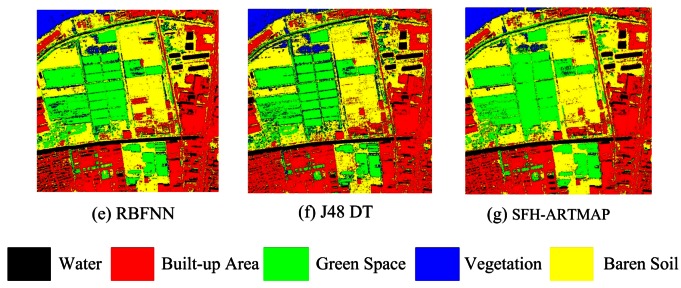
Classification results of using only spectral features.

**Figure 6.**
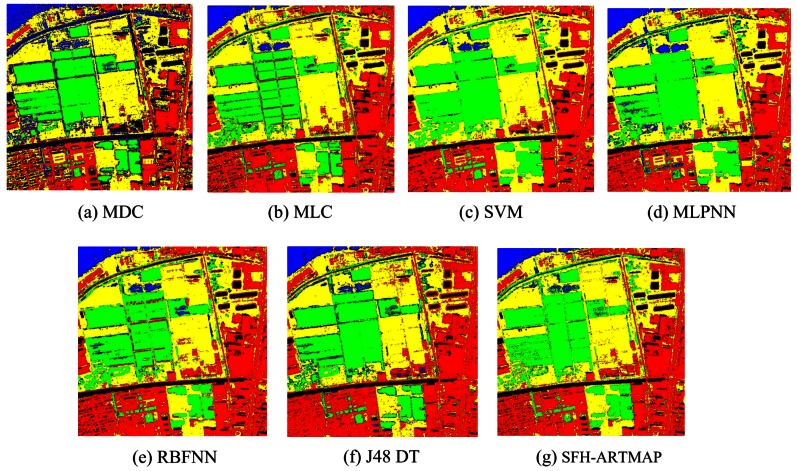
Classification results of using both spectral and textural features.

In addition to original multispectral image, textural features extracted from the Band 4 which has the richest information are also added for classification. Mean and variance extracted by gray level co-occurrence matrix (GLCM) are used to describe the textural features. Two schemes are used for classification. The first scheme is only using the spectral features of four multispectral bands as the inputs, and the second scheme is using both four spectral features and two textural features as the inputs. Seven classifiers are used, including: MMDC, MLC, SVM, MLPNN, RBFNN, J48 DT, and SFH-ARTMAP. The accuracies of all classifiers using only spectral features and spectral together with textural features are listed in Tables 2 and 3. The classification result can be seen in Figures 5 and 6.

Comparing the test areas, the accuracy assessment demonstrates that all the classifiers perform very accurately and achieve overall accuracies of 85.92% (MMDC), 92.78% (MLC), 93.41% (SVM), 93.49% (MLP), 92.83% (RBFNN), 92.42% (J48 DTC) and 93.15% (SFH-ARTMAP) using spectral and texture features (seen Table 3). As expected, the kappa values of the classification results using spectral and texture features are slightly higher than the ones only using spectral features. The specific accuracies of built-up areas of MMDC, MLC, MLPNN and SFH-ARTMAP are significantly lower (less than 79%). Using spectral and texture features together, most obvious is that the accuracies of built-up areas are increased by up to 7.8%, 8.14%, 8.72%, 9.44% of MMDC, MLC, MLPNN and SFH-ARTMAP, respectively. Furthermore, some other findings can be summarized as follows:

1. When textural features are introduced as the input of the classifiers, both the overall and class accuracies are enhanced in the most cases, especially for built-up areas, demonstrating the superiority of incorporating textural features in the classification process.
2. The classifiers have shown the different performance on the specific classes, indicating that the classifier performing well for one class may be poor for other classes. For instance, when only using spectral feature, MMDC performs weaker on the land cover type of “built-up areas” (48.48%) but SVM achieve the accuracy of 97.51% (Table 2). The situation for spectral and texture features is similar to the one for spectral feature. The highlights in Tables 2 and 3 are the highest accuracy of class among the classifiers. From the above analysis, it is necessary to combine multiple classifiers to find a better result than any individual classifiers. In the following experiments on QuickBird image, the classification results derived from both spectral and texture features are adopted as the individual classification results.

**Figure 7.**
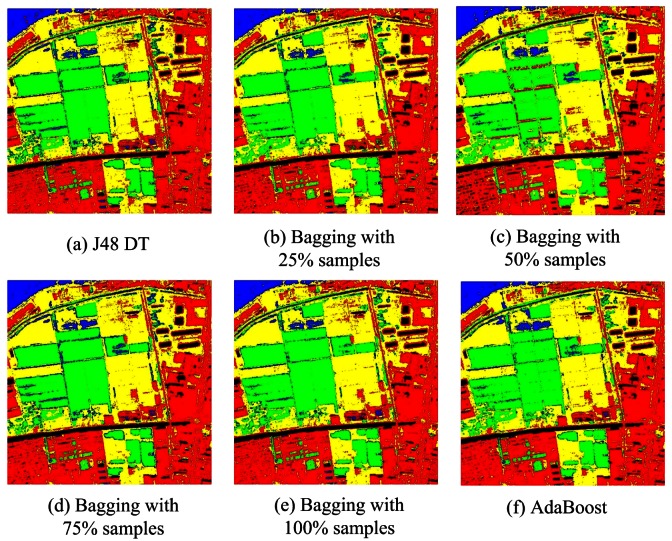
Classifications of J48 DTC Based on Bagging and AdaBoost.

**Figure 8.**
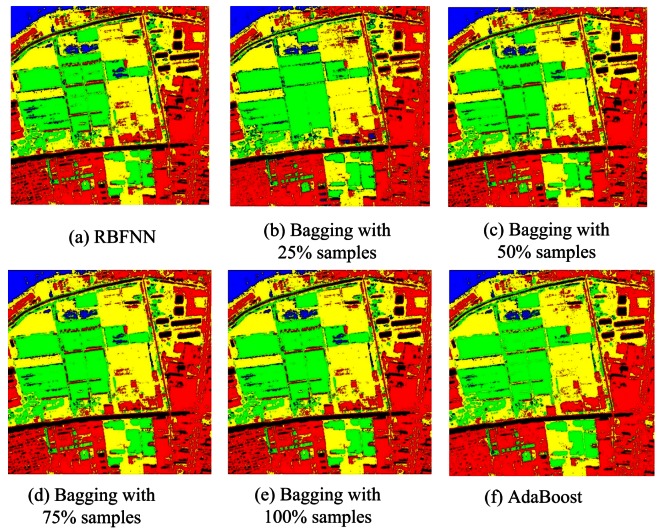
Classifications of RBFNN Based on Bagging and AdaBoost.

**Figure 9.**
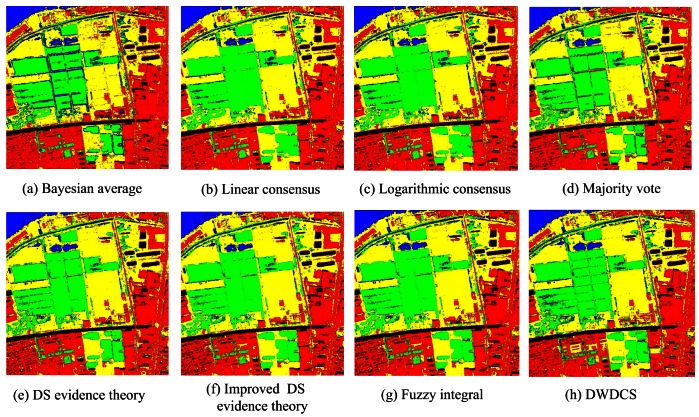
Classification results based on parallel combination approaches.

For Bagging and AdaBoost, J48 DTC, RBFNN and MLPNN are used as the base classifiers, and the accuracies are summarized in Table 4. Both Bagging and AdaBoost obtain very accurately results and achieve very high overall accuracies (94.93% and 95.67% for J48 DTC, 95.09% and 95.43% for MLPNN, 93.36% and 95.35% for RBFNN). On the other hand, the accuracies of Bagging and AdaBoost increased by 0.89% to 1.96% and 1.94% to 2.25% for the three base classifiers. The classification maps can be seen in Figures 7–9. The theories of Bagging illustrate that it shows excellent performance when the base classifier is an unstable classifier such as decision tree, neural networks *et al*. [16]. In most cases, the performance of AdaBoost is superior to Bagging. Our experimental results are compatible with the theoretical analysis. In addition, we also studied the influence of bootstrapped sample size to classification accuracy. Here, we choose four sample sizes: 25%, 50%, 75% and 100%. The general trend is that the overall accuracies increase slightly (less than 1%) when the sampling rate rise, although the accuracy of RBFNN is a bit unsteady under different sampling rate. Perhaps the reason is that the iterations of RBFNN are not enough (only 10 in the experiment). The results indicate that Bagging and AdaBoost outperform the base classifier in terms of overall accuracy.

In order to illustrate the effectiveness and universality of MCS, we choose different member classifiers applied to different parallel combination rules. For the parallel combination methods such as Bayesian average, logarithmic consensus and linear consensus, the member classifiers are MLC, SVM, MLPNN, RBFNN and SFH-ARTMAP. The member classifiers for others are SVM, MLPNN, RBFNN, MEDC and J48DT. Figure 9 presents the classification results of MCS on parallel combination, and their accuracy statistics are shown in Table 5. Among the individual classifiers, SVM classifier achieves the highest accuracy of 93.49%. Compared to SVM classifier, Bayesian average achieves the accuracy of 94.99%, with the improvement of 1.5%; Majority vote and fuzzy integral improve the accuracy from 93.49% to 94.74% and 93.49% to 94.37%, respectively. Other parallel combination approaches improve the accuracy slightly with the improvement of 0.28% to 1%. The “local” ensemble method (DWDCS) results in 93.98% accuracy with the improvement of 0.49%. Compared with the overall accuracies, the kappa coefficients show similar characteristics. Bayesian average obtains the highest accuracy for all parallel combination schemes.

Unlike other combination algorithms, DWDCS pays attention to the local optimum while other combination algorithms achieve the global optimum. The computation time of DWDCS is proportional to the test sample size and the nearest neighbors. In our experiments, the test sample size is more than 500 and the nearest neighbor is selected to be 15. The computation time of DWDCS is more than 10 min and other parallel combination strategies are less than 20 s.

**Figure 10.**
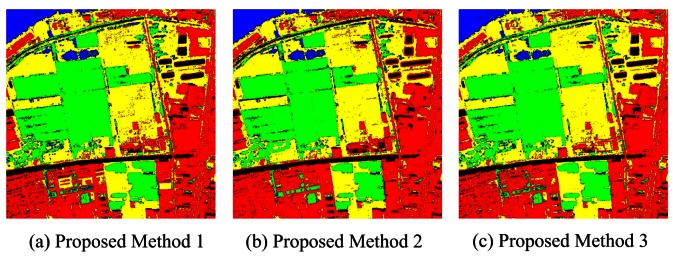
Classification results based on proposed concatenation combination algorithms.

Figure 10 presents the classification result of MCS based on concatenation combination and Table 6 shows the accuracy statistics. The accuracy assessment indicated that all the proposed concatenation combination algorithms perform very accurately and obtain overall accuracies of 95.21% (method 1), 97.06% (method 2) and 95.05% (method 3). Among all the classifier ensemble approaches in MCS, the proposed method 2 obtains the highest accuracy (97.06%) due to the following reasons:

1. This proposed method adopts the probabilities as the input of the second level and there is obvious diversity among these probabilities;
2. The output distributions of different classifier are different, for instance, the probability distribution of SVM and MLP are focused on 0 or 1, while the probability distribution of MEDC is ranged from 0.45 to 0.55. The linear combination rules such as linear consensus are not suited, and SVM can tackle non-linear problems to a good extent. Therefore, it can achieve the highest accuracy compared with others.

In the experiment of diversity measures, we choose five member classifiers from the classifier pool to be combined based on diversity measure. The classifiers in the pool are MLPNN (1), SVM (2), SFH-ARTMAP (3), RBFNN (4), MLC (5), J48DT (6), MMDC (7). Thus, there are twenty-one selection schemes of five classifiers. We have calculated the diversity of the all combination. The optimum combination corresponding to the diversity measure can be seen in Table 7. Then, we adopted the different parallel combination approaches (majority voting, weighted voting, fuzzy integral, DS evidence theory) to evaluate the performance of the combination of classifiers selected by diversity measures.

From Table 7, *k, k*′, *entropy* and *WCEC* diversity measures selected the same combination, which contains the classifiers: MLPNN, SVM, SFH-ARTMAP, J48DT, MMDC. *DF* selected the combination of MLPNN, SFH-ARTMAP, RBFNN, J48 DTC and MMDC. MLPNN, SFH-ARTMAP, RBFNN, MLC, J48DTC and MMDC were chosen by *DA*. The overall accuracies of the combination selected by *DA* are 95.52% (majority vote), 95.39% (weighted vote), 95.40% (fuzzy integral) and 95.36% (DS evidence theory). In contrast, the combination selected by *DF* results in the accuracies of 95.05% (majority vote), 94.79% (weighted vote), 94.76% (fuzzy integral) and 94.86% (DS evidence theory). The combination selected by other four measures achieves the accuracies of 94.83% (majority vote), 93.54% (weighted vote), 93.77% (fuzzy integral) and 93.55% (DS evidence theory). Comparison of these results with the accuracy assessment indicates that the combination selected by *DA* outperform the ones selected by other diversity measures in terms of overall accuracy. It demonstrates that the proposed diversity measure is a positive guidance for classifier combination. Furthermore, we ranked all the combination according to the *DA* measure in Table 8 and the highlights are the first three ones, whose accuracy produced by the parallel combination approaches can be seen in Table 7.

### 3.2. Experiment 2: OMISII Hyperspectral Remote Sensing Image

**Figure 11.**
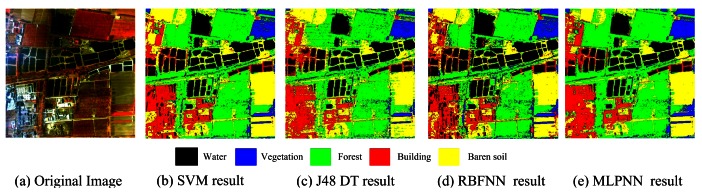
False color composite of OMIS image and classification results of individual classifier.

Original airborne OMISII hyperspectral remote sensing image has 64 bands with spectral range: 0.45–1.09 *μm*. The spectral resolution is 10 *nm*. In this paper, Zhongguangcun, a high-tech zone of Beijing City is chosen as the study area. The five noisy bands are removed so it is remaining 59 bands for classification. The image size is 400 × 400. Figure 11(a) is the false color composite of the image by using Band 27, 25 and 2 as R, G and B components. Training samples and test samples are selected independently from the image. The land cover is classified into five classes: water, building, vegetation, forest and bare soil. From Table 9, SVM classifier has the highest classification accuracy of forest (92.69%) and bare soil (92.97%); MLPNN classifier produces the highest classification accuracy of vegetation (98.10%) and building (98.34%); RBFNN generates the highest classification accuracy of water (95.72%). These results indicate the diversity among classifiers, which is similar to the experiment on QuickBird image. MLPNN achieves the highest overall accuracy of 92.53%. Figure 11(b-e) shows the classification results of SVM, J48 DTC, RBFNN and MLPNN respectively. Based on diversity measure among all classifier combination schemes, MMDC, MLPNN and SVM classifier are selected to generate the final classification.

**Figure 12.**
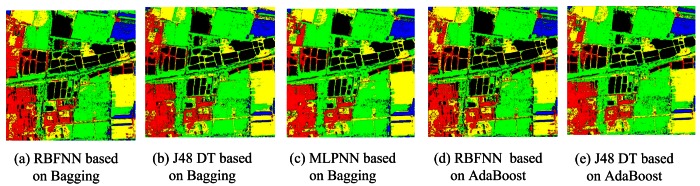
Classification result based on AdaBoost and Bagging.

**Figure 13.**
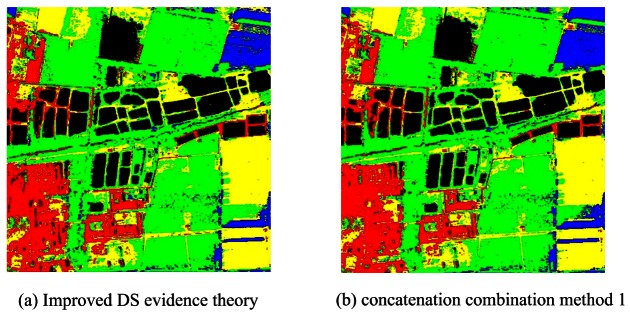
Classification result using parallel and concatenation combination style.

From Table 10, both AdaBoost and Bagging can enhance the classification accuracy of the base classifier. Bagging can improve the accuracy from 87.86% to 90.44% for J48 DTC, 89.46% to 89.88% for RBFNN and 92.53% to 93.09% for MLPNN. In contrast, AdaBoost performs a bit better (91% for J48 DTC, 90.65% for RBFNN and 93.27 for MLPNN) than bagging when the same base classifier is used. Figure 12 are the classification results based on AdaBoost and Bagging using RBFNN, MLPNN, J48 DTC as the base classifiers. In classification ensemble based on parallel/concatenation combination, both improved D-S evidence theory and concatenation combination (proposed method 1) can obtain very accurately classification result with the accuracies of 92.65% and 93.58% than the best individual classifier (92.53%). This demonstrates that these methods are effectively to improve the accuracy. Figure 13(a) is the result of improved D-S evidence theory using MMDC, MLPNN and SVM as member classifiers. Figure 13(b) is the result of concatenation combination (proposed method 1).

### 3.3. Experiment 3: Medium Resolution Multi-Spectral Landsat ETM+ Image

A Landsat ETM+ image acquired on September 17th 2004 is used to validate the above methods. The used bands of Landsat ETM+ images are as follows: band 1: Blue band; band 2: Green band; band 3: Red band; band 4: NIR band; band 5: MIR band, band 7: LIR band. The image size is 500 × 500. The study area is the central part of Xuzhou City, China. Through the image analysis and field works, the land cover is classified into five classes: water, farmland, woodland, built-up land, and public green space. We use five member classifiers: RBFNN, J48DT, MMDC, MLC and MEDC. The adopted classifier combination methods are majority voting, weighted voting, D-S evidence theory, and fuzzy integral.

**Figure 14.**
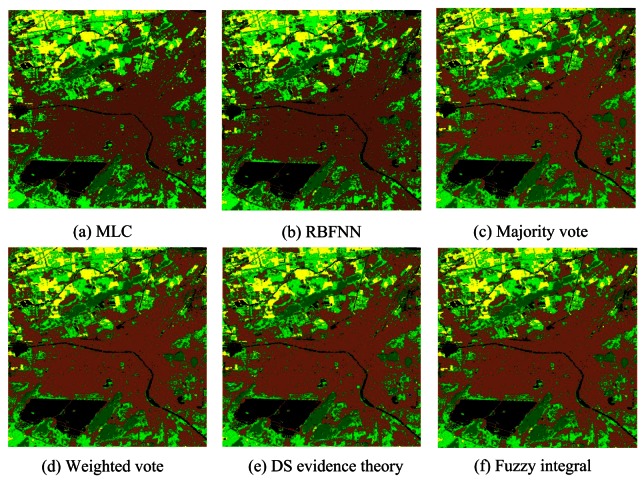
Classification results of Landsat ETM+ image using different schemes.

Table 11 summarizes the accuracies of those classification results. MLC achieves the highest accuracy (91.19%). All the four classifier ensemble methods obtain higher overall accuracies than MLC classifier. The accuracy of majority vote increases from 91.19% to 93.55%. Weighted vote achieves the accuracy of 93.55%, with the improvement 2.36%. The accuracy is increased by up to 2.23% and 1.8% by DS evidence theory and fuzzy integral approaches. This indicates that these methods effectively improve the accuracy. Figure 14 presents the classification results of different classifiers and classifier combination strategies.

### 3.4. Discussion

Based on the above analysis, we identified that the incorporation of diversity among the classifiers is of great importance in order to obtain better classification results via a MCS [31]. Combining the similar classification results would not further improve the overall accuracy. Bagging uses bootstrap sampling to generate diversity [16]. In order to enhance the diversity, concatenation combination constructs a proper dataflow so that diverse decisions could be first made by a subset of the committee classifiers and their conclusions are then set to another subset of classifiers. It is worthy to point out that the most important issue to combine the classifiers in parallel combination method is to examine the difference among them using diversity measures. In addition, the selection of an appropriate ensemble strategy is also an important issue of MCS. This paper presents a comprehensive list of the most common parallel combination approaches and also analyzes the situation in which each of them are most useful. We also experimentally prove the effectiveness of the three modified concatenation combination algorithms within certain images. Furthermore, we studied the diversity measures to select the optimum combination. Compared to other diversity measures, the proposed diversity measure considers both the difference and accuracy among the classifiers. Thus, it can select the optimum combination whose accuracy is higher than the selected ones by other measures.

## 4. Conclusions

This review discusses the intensive contributions of MCS-based work in remote sensing. We present a comprehensive review of the most popular approaches and analyze the situations in which each of them is most useful. Those algorithms are experimented with three typical remotely sensed images, high resolution QuickBird image, airborne hyperspectral OMISII image, and medium Landsat ETM+ image. Some modified algorithms are proposed based on the ideas of improving MCS performance, and we experimentally proved the usefulness of these proposed methods.

Most of the findings from this paper show that both concatenation and parallel combination can enhance classification accuracy, but their performances are affected by different factors such as selected member classifiers, classifier combination criterion, *etc.* Furthermore, according to our experimental results, diversity measures can play active guidance for the selection of multiple classifiers combination.

## Figures and Tables

**Table 1.** Summary of the classifier ensemble approaches in parallel combination.

**Name**	**hard labels**	**soft labels**	**validation set**
Majority vote	Y	N	N
Weighted vote	Y	N	Y
Bayesian average	N	Y	N
Dempster-shafer evidence theory	Y	N	Y
Fuzzy integral	Y	N	Y
Consensus theory	Y	Y	Y
Dynamic classifier selection	Y	N	Y

* Note: “Y” and “N” mean whether or not the hard labels, soft labels or validation set are needed. Dynamic classifier selection method needs the original image to calculate the distance.

**Table 2.** Classification accuracy of the first Scheme (only spectral features).

**Classifier**	**Water**	**Built-up Area**	**Green Space**	**Vegetation**	**Barren Soil**	**OA**	**Kappa**
MMDC	**96.81**%	48.48%	92.18%	95.64%	97.68%	84.41%	0.81
MLC	95.17%	75.00%	96.54%	92.01%	**98.01**%	90.80%	0.88
SVM	87.56%	**97.51**%	**95.73**%	89.05%	92.77%	**92.59**%	**0.91**
MLPNN	92.12%	78.04%	96.98%	**95.04**%	97.80%	91.44%	0.89
RBFNN	94.23%	80.59%	95.25%	87.84%	96.10%	90.65%	0.88
J48 DTC	93.85%	89.19%	89.19%	86.75%	92.78%	91.92%	0.90
SFH-ARTMAP	92.77%	77.85%	**97.25**%	91.23%	96.39%	90.70%	0.88

**Table 3.** Classification accuracy of the second scheme (spectral and textural features).

**Classifier**	**Water**	**Built-up Area**	**Green Space**	**Vegetation**	**Barren Soil**	**OA**	**Kappa**
MMDC	88.08%	56.28%	93.61%	**97.16**%	96.76%	85.92%	0.82
MLC	95.59%	83.14%	97.11%	91.23%	98.34%	92.78%	0.91
SVM	90.56%	**98.62**%	97.39%	84.33%	94.51%	93.41%	0.92
MLPNN	**97.60** %	86.76%	95.12%	88.87%	**98.84**%	**93.49**%	**0.92**
RBFNN	94.93%	88.66%	93.34%	89.35%	97.80%	92.83%	0.91
J48 DTC	92.82%	93.23%	95.69%	86.45%	96.51%	92.42%	0.90
SFH-ARTMAP	93.34%	87.29%	**97.56**%	90.87%	**98.84**%	93.15%	0.91

**Table 4.** Accuracy of AdaBoost and Bagging using different base classifier.

**Base classifier**	**J48 DTC**	**MLPNN**	**RBFNN**
Base classifier	92.42%	93.49%	92.83%
Bagging(25% samples)	94.22%	94.20%	93.76%
Bagging(50% samples)	94.55%	95.27%	93.39%
Bagging(75% samples)	94.30%	94.91%	93.40%
Bagging(100% samples)	94.93%	95.09%	93.36%
AdaBoost	95.67%	95.43%	95.35%

**Table 5.** Classification accuracy of parallel classifiers combination

**Combination strategy**	**OA**	**Kappa**
Bayesian average	94.99%	0.94
Logarithmic consensus	93.77%	0.92
Linear consensus	94.37%	0.93
Majority vote	94.74%	0.93
Evidence theory	93.76%	0.92
Improved evidence theory	94.18%	0.93
Fuzzy integral	94.37%	0.93
DWDCS	93.98%	0.92

**Table 6.** Classification accuracies of concatenation combination algorithms.

**Concatenation combination**	**OA**	**Kappa**
Proposed method 1	95.21%	0.94
Proposed method 2	97.06%	0.96
Proposed method 3	95.05%	0.94

**Table 7.** The optimum combination selected by diversity measures.

**Diversity**		**Classifiers combination**	**Majority vote**	**Weighted majority vote**	**Fuzzy integral**	**DS Evidence theory**
*k*		1,2,3,6,7	94.83%	93.54%	93.77%	93.55%
k′		1,2,3,6,7	94.83%	93.54%	93.77%	93.55%
DF		1,3,4,6,7	95.05%	94.79%	94.76%	94.86%
Entropy		1,2,3,6,7	94.83%	93.54%	93.77%	93.55%
WCEC		1,2,3,6,7	94.83%	93.54%	93.77%	93.55%
DA	1	1,3,4,5,6	95.52%	95.39%	95.40%	95.36%
	2	1,3,4,6,7	95.05%	94.79%	94.76%	94.86%
	3	1,3,4,5,6	95.32%	95.15%	95.22%	95.20%

**Table 8.** Results of DP measure of all five classifiers' combination.

**Classifiers combination**	**DP**	**Rank**	**Classifiers combination**	**DP**	**Rank**
1,2,3,4,5	1.68520	10	1,3,4,5,7	1.74182	6
1,2,3,5,6	1.69234	8	**1,3,5,6,7**	**1.75065**	**3**
1,2,3,5,7	1.67308	16	**1,3,4,6,7**	**1.75104**	**2**
1,2,3,4,6	1.69413	7	1,4,5,6,7	1.74351	5
1,2,3,4,7	1.67177	18	2,3,4,5,6	1.68378	11
1,2,3,6,7	1.68283	12	2,3,4,5,7	1.66168	21
1,2,4,5,6	1.68565	9	2,3,5,6,7	1.67331	15
1,2,4,5,7	1.66347	19	2,3,4,6,7	1.67285	17
1,2,5,6,7	1.67524	13	2,3,5,6,7	1.66311	20
1,2,4,6,7	1.67524	13	3,4,5,6,7	1.74569	4
**1,3,4,5,6**	**1.75277**	**1**			

**Table 9.** Classification accuracy of single classifier.

**Classifier**	**Water**	**Building**	**Forest**	**Vegetation**	**Bared soil**
SVM	80.57%	97.34%	**92.69**%	89.96%	**92.97**%
J48DT	88.07%	86.38%	88.80%	95.82%	80.92%
RBFNN	**95.72**%	88.70%	89.58%	90.87%	81.63%
MMDC	94.80%	96.35%	88.42%	95.82%	80.92%
MLPNN	87.77%	**98.34**%	90.35%	**98.10**%	88.69%

**Table 10.** Classification accuracy statistics.

**Method**	**OA**	**Kappa**
SVM	90.72%	0.88
J48 DTC	87.86%	0.85
RBFNN	89.46%	0.87
MMDC	91.42%	0.89
MLPNN	92.53%	0.91
RBFNN based on bagging	89.88%	0.87
MLPNN based on bagging	93.09%	0.91
J48 DT based on bagging	90.44%	0.88
RBFNN based on AdaBoost	90.65%	0.88
J48 DT based on AdaBoost	91.00%	0.89
MLPNN based on AdaBoost	93.27%	0.91
Improved DS evidence theory	92.95%	0.91
Concatenation combination	93.58%	0.92

**Table 11.** Classification accuracies of Landsat ETM+ image.

**Method**	**OA**	**Kappa**
RBFNN	88.09%	0.85
MMDC	86.35%	0.83
MLC	91.19%	0.89
MEDC	80.40%	0.76
J48 DTC	87.84%	0.85
Majority vote	93.55%	0.92
Weighted vote	92.56%	0.91
DS evidence theory	93.42%	0.92
Fuzzy integral	92.99%	0.91
